# Awareness of Being at Risk of Stroke and Its Determinant Factors among Hypertensive Patients in Banyumas, Indonesia

**DOI:** 10.1155/2022/4891134

**Published:** 2022-04-11

**Authors:** Ismail Setyopranoto, Arif Setyo Upoyo, Atyanti Isworo, Yunita Sari, Amelia Nur Vidyanti

**Affiliations:** ^1^Department of Neurology, Faculty of Medicine, Public Health, and Nursing, Universitas Gadjah Mada, Yogyakarta 55281, Indonesia; ^2^Department of Nursing, Faculty of Health Sciences, Universitas Jenderal Soedirman, Purwokerto, Central Java 53122, Indonesia

## Abstract

**Background:**

The global burden of stroke is still high, particularly in developing countries, with hypertension serving as the main risk factor. The awareness of stroke among hypertensive patients is crucial for stroke prevention. This study was aimed at identifying the awareness of being at risk of stroke and its determinant factors among hypertensive patients in Banyumas, Indonesia.

**Methods:**

This was a cross-sectional study conducted in Primary Health Centers (PHCs) in Banyumas Regency, Indonesia, from April to August 2019. A simple random sampling technique was used to select the representatives' PHCs. Hypertensive patients who came regularly to the PHCs were consecutively recruited. Data were collected through a self-reported questionnaire and medical records. The main outcome was the level of participants' awareness of being at risk of stroke. Multivariate logistic regression analysis was performed to measure determinant factors associated with the level of awareness.

**Results:**

Out of 457 patients investigated, 77.46% had a low level of awareness. Low knowledge of hypertension, low income, and no history of previous stroke were associated with a low-level of awareness (odds ratio (OR) 1.942, 95% CI 1.195-3.158, *p* 0.007; OR 2.321, 95% CI 1.326-4.064, *p* 0.003; and OR 6.033, 95% CI 2.450-14.858, *p* < 0.001, respectively).

**Conclusion:**

Majority of hypertensive patients were unaware of being at risk of stroke. Knowledge of hypertension, income, and history of previous stroke are factors that may influence the awareness of being at risk of stroke among them. This emphasizes the need to provide better campaigns and education programs to raise the awareness of stroke in a community setting, particularly for the population at risk.

## 1. Introduction

Stroke is the leading cause of morbidity and disability worldwide [1]. The global burden of stroke is still high with the estimated incidence of 15 million new cases per year, in which two-thirds of them occur in developing countries [2]. The disability-adjusted life years of stroke patients was more than 87% in developing countries, and this was seven times higher than in developed countries [3, 4].

Indonesia has the highest rates of age-sex standardized stroke mortality and disability adjusted life-years related to stroke among South-East Asian (193.3/100,000 person-years and 3,382.2/100,000 people, respectively) [5]. The average age of stroke patients in Indonesia is lower than that in developed countries [6]; this leads to a higher disability rate, reduced the quality of life, and more economic lost [7]. The high burden of stroke is attributable to modifiable risk factors such as behavioral, metabolic, and environmental risk factors [8]. Hence, knowledge related to stroke warning signs and risk factors is crucial to reduce the incidence of stroke and prevent subsequent mortality [9].

Hypertension is the most common risk factors for stroke and the main modifiable factor attributed to disability after stroke [10, 11]. The Indonesia Family Life Survey-5 (IFLS-5) reported that the prevalence of hypertension in people with the mean age of 43.3 years old was quite high, reaching 33.4%. In addition, the awareness of hypertension was low, and the treatment and follow-up were inferior [12]. This condition can be a leading risk factor for high stroke prevalence in Indonesia. Furthermore, based on the Indonesian Basic Health Research 2018, the stroke prevalence had been increasing from 7/1000 population in 2013 to 10.9/1000 population in 2018 [13]. The increasing prevalence of stroke in Indonesia may be caused by low stroke awareness. A previous study conducted in Indonesia showed that the majority (65.7%) of hypertensive patients in Indonesia had poor stroke prevention behavior. The most dominant factor contributing to poor stroke prevention behavior was the awareness of stroke [14]. However, this study mostly focused on factors influencing stroke prevention behavior. They did not explore more detail about the determinant factors related to the awareness of stroke.

Raising public of stroke awareness would be beneficial for better stroke prevention strategies, particularly for the population at risk. Approximately 80% of new stroke incidence could be avoided by improving knowledge related to risk factors of stroke [15]. The knowledge about stroke as a complication of hypertension is also essential for establishing effective prevention programs. Although the prior study had revealed that stroke awareness mostly contributed to poor stroke prevention behavior among hypertensive patients [14], little is known about the variables that influence the level of awareness of stroke, particularly in Indonesia. Therefore, we aimed to identify awareness of being at risk of stroke and its associated factors among hypertensive patients in Banyumas, Indonesia.

## 2. Materials and Methods

### 2.1. Study Design and Participants

This study used a cross-sectional design. The population of this study was hypertensive patients who came regularly to Primary Health Centers (PHC) in Banyumas Regency to do a routine check-up every month. There are 39 PHCs in Banyumas Regency, in which their locations are dispersed over a large area. Due to the difficulties in reaching the faraway locations and limited budget, we decided to recruit the participants from 5 PHCs as representatives. Simple random sampling using a computer-generated random number (GraphPad QuickCalcs, GraphPad Software Inc., La Jolla, CA, USA) was performed to select 5 out of 39 PHCs. These five PHCs are located in rural areas (3 PHCs) and urban areas (2 PHCs).

Subsequently, participants were recruited in those 5 PHCs consecutively during April-August 2019 until a minimum sample was achieved. The inclusion criteria were hypertensive patients aged >35 years old who came to the PHCs for a routine checkup. Hypertensive patients with comorbidities such as aphasia, dementia, or other neuropsychiatric diseases were excluded from the study.

We estimated the minimum sample size and found that it might be acceptable to represent the entire population in Banyumas Regency. The sample size was calculated using the following formula for cross-sectional studies [16]:

(1) $$\begin{array}{c} \frac{N={Z\alpha }^{2}\times P\times Q}{d^{2}}, \end{array}$$

where *N* is the sample size, *Zα* is the statistic corresponding to the level of confidence, *P* is the expected prevalence (that can be obtained from the same studies or a pilot study conducted by the researchers), *Q* = 1–*P*, and *d* is the precision (corresponding to effect size).

Based on the previous study [17], the proportion of patients who have good knowledge of stroke risk factors was 53.50%. With *Zα* = 1.96 and *d* = 0.05, the minimum sample size is as follows:

(2) $$\begin{array}{c} \frac{N=\;{Z\alpha }^{2}\times P\times Q}{d^{2}},\\ \frac{N={\left(1.96\right)}^{2}\times 0.535\times 0.465}{0.05^{2}},\\ N=383\text{ participants}. \end{array}$$

By adding a 15% nonresponse rate, the final sample size was 441. A total of 457 participants responded completely in this study, with an average of 91 patients recruited from each PHC. All patients signed a written informed consent prior to the investigation.

### 2.2. Data Collection and Measurements

We conducted a cross-sectional survey by interviewing the participants based upon a questionnaire. The questionnaire for the independent variables consists of three components, including demographic characteristics, knowledge on hypertension, and stroke risk factors.

Demographic characteristics included sex, age, education level, marriage status (married/widow), living with (family/alone), and income. Income was categorized as <minimum regional payment (MRP) or <134 USD/month, and ≥MRP (≥134 USD/month) based on the minimum standard income in Indonesia [18] and history of headache.

We measured patients' knowledge on hypertension as a risk factor for stroke by using the Hypertension Knowledge-Level Scale (HKLS). HKLS has high validity and reliability for measuring the level of knowledge on hypertension in adults [19]. It has been translated and validated to the Indonesian version with good reliability [20]. The final scale has 22 items related to hypertension which are divided into six subdimensions, namely, definition, medical treatment, drug compliance, lifestyle, diet, and complications. The maximum score is 22 for the entire scale. The minimum score is zero for the entire scale and for all subdimensions [19]. HKLS was further classified as low knowledge on hypertension (HKLS ≤ 17) and high knowledge (HKLS 18-22) [21].

Stroke risk factors included medication adherence, history of hypertension (controlled/uncontrolled), diabetes mellitus, history of dyslipidemia, history of previous stroke, history of atrial fibrillation, history of heart diseases, history of anxiety, and history of sleep apnea. The medical history was investigated by a questionnaire with “yes” and “no” answers. However, we also inquired about their medical records to validate the answers.

Medication adherence was measured by a self-reported questionnaire related to the blood pressure medicine, consisting of five questions [22, 23]. The patients had to respond with “yes” or “no” to the following questions: “Over the past week, have you taken your blood pressure medication as you should on schedule?” Do you ever forget to take your medicine?” “Are you careless at times about taking your medicine?” When you feel better do you sometimes stop taking your medicine?” and “Sometimes if you feel worse when you take the medicine, do you stop taking it?” If the responses were “no” to all 5 questions, the patients were categorized as “adherent.” If the response was “yes” to one or more questions, the patients were categorized as “nonadherent” [22, 23].

For the dependent variable, we measured the awareness of being at risk of stroke among the participants by a self-reported question modified from previous study [24]: “How sure are you that you are currently at risk of having a stroke?” The responses were documented by using a Likert scale (1-5). Scale 1 means the participants were strongly not sure of being at risk of stroke, scale 2 means not sure, scale 3 means undecided, scale 4 means sure, and scale 5 means very sure. We further categorized the level of awareness based on the prior study [14]: participants with a Likert scale ≤ 3 were categorized as having a low level of awareness, and those with a Likert scale > 3 were categorized as having a high level of awareness.

### 2.3. Statistical Analysis

We performed the chi-square test to analyze the differences between categorical variables in the baseline characteristics. Factors associated with the awareness of stroke were measured using univariate logistic regression analysis. Subsequently, these associated factors were further analyzed using multivariate logistic regression to measure their contributions to the level of stroke awareness after controlling the covariates. Statistical significance was indicated by a *p* value of <0.05 and odds ratio (OR). SPSS version 16 was used for all the analyses.

## 3. Results

### 3.1. Baseline Characteristics

We involved 457 patients with hypertension. Most of them were female (80.7%), were aged >55 years old (75.7%), had low education level (79.6%), had low HKLS (56%), were married (71.8%), were living with family (51%), and had low income (67.1%). Three hundred and fifty-four (77.46%) of the 457 patients had low awareness of stroke, whereas 103 (22.54%) had high awareness of stroke (Table 1). Factors associated with the awareness of stroke were HKLS (odds ratio (OR) 1.902, 95% CI: 1.221-2.963, *p* 0.004) and income level (OR 2.156, 95% CI: 1.374-3.383, *p* 0.001).

### 3.2. Association between Stroke Risk Factors and the Awareness of Stroke

Table 2 presents the association between stroke risk factors with the awareness of stroke. Majority of participants had nonadherence medication (59%), had uncontrolled hypertension (85.33%), and had no history of diabetes mellitus, hyperlipidemia, stroke, atrial fibrillation, heart diseases, and sleep apnea. History of hypertension, DM, and history of previous stroke were significantly associated with the awareness of stroke (OR 0.536, 95% CI: 0.305-0.944, *p* 0.029; OR 1.970, 95% CI: 1.264-3.071, *p* 0.003; and OR 4.504, 95% CI: 1.953-10.390, *p* < 0.001, respectively).

### 3.3. Multivariate Analysis of Factors Associated with a Low Awareness of Stroke

Table 3 presents the multiple logistic regression of factors associated with the awareness of stroke after adjustment by controlling multiple covariates. We found that knowledge of hypertension, income, and a history of previous stroke were independently associated with the awareness of stroke among hypertensive patients. Participants with low knowledge of hypertension, low income, and no history of prior stroke were more likely to have a low level of stroke awareness. History of previous stroke was the most dominant factor which influenced the awareness (OR 6.033, 95% CI: 2.450-14.858, *p* < 0.001).

## 4. Discussion

The study shows that knowledge of hypertension, income, and a history of previous stroke were associated with the awareness of stroke. The majority of the participants had poor awareness of stroke. To our knowledge, this study is the first study in Indonesia which demonstrated that hypertensive patients with low knowledge of hypertension, low income, and no history of previous stroke were more likely to be unaware that they were being at risk of stroke.

This finding is in accordance with prior studies which showed that most participants were not fully aware of stroke and its risk factors [15, 25, 26]. Moreover, the low level of awareness in the present study was positively associated with poor knowledge of hypertension. This may endanger their health status as patients with poor knowledge could not prevent the future risk of stroke.

Corroborating with our findings, a previous study reported that 78.3% of patients had inadequate knowledge, 21.7% had moderate knowledge, and none of them had adequate knowledge on hypertension and stroke prevention [27]. Two previous studies investigating the knowledge about stroke warning signs and risk factors in patients with acute cerebrovascular disease [28] and a history of previous stroke [29] revealed similar findings. Another cross-sectional study on hypertensive patients also demonstrated that 75.1% of participants had poor knowledge of stroke prevention [30]. Knowledge of hypertension is crucial to prevent the increasing incidence of hypertension and to maintain a normal blood pressure control [31]. Hence, knowledge deficits contribute to inadequate control of hypertension and poor medication adherence [21], which further increase the risk of stroke. In addition, people with higher levels of education were more likely to seek emergency assistance if they were being at risk of stroke [15, 17, 32]. The lack of knowledge related to stroke further increases the delay in visiting a health center [17, 32]. This highlights that low knowledge is associated with low stroke awareness.

In the study, we also found that low income was positively associated with stroke risk awareness. This finding is supported by a prior study from the 2017 National Health Interview Survey in the United States which showed that individuals with low income were more likely not to be aware of all stroke symptoms and risk factors [33]. Another study reported that people with low income were more likely to have a lower willingness to take a stroke patient to a hospital than those with high income. The reason may be attributed to low education and low knowledge related to stroke risk factors and warning signs [15]. This is comparable with previous study which found that the level of monthly income was a factor that could predict stroke knowledge. Higher income was associated with a good stroke knowledge [34]. Moreover, another cross-sectional study in Spain demonstrated that socioeconomic status including educational level, income, and employment status are independent factors for sufficient knowledge of stroke [35]. Patients with higher income are more likely to have better and prompt health-seeking behavior when they are being at risk of stroke [36].

The awareness of being at risk of stroke in this study was also influenced by having a history of stroke. In accordance with our finding, a cross-sectional study among stroke patients in Spain reported that history of prior stroke was associated with higher knowledge on stroke warning signs and risk factors [17]. Another study showed that stroke survivors or patients with a history of previous stroke were more likely to have more awareness of stroke warning signs including sudden numbness or weakness, sudden difficulty speaking or understanding speech, sudden dizziness, and sudden severe headache [37]. The contributing factor for higher stroke awareness among patients with a history of prior stroke is the adequate information received during their stay in the hospital, from admission until discharge. This permits them to recognize the signs, symptoms, and risk factors of stroke which could prevent further delay in seeking care [17, 29, 38]. Therefore, having no history of previous stroke could contribute to having low knowledge of stroke risk factors and the prevention methods or low stroke awareness.

Hypertension and diabetes mellitus (DM) are established risk factors of stroke. In the present study, we found no association between prior history of hypertension and DM with the awareness of stroke. Our findings corroborate the previous study which demonstrated that a history of hypertension and DM was not associated with knowledge on stroke [17]. Contradictory to our findings, another study found that having a long duration of hypertension was associated with good knowledge on stroke prevention methods [30]. Notably, this difference may be due to different methods. Further research is warranted to delineate this relationship.

Results in the present study contribute to providing more evidence that stroke awareness is still insufficient in the community setting. This indicates an urgent need to disseminate stroke education and awareness campaigns in Indonesia, particularly targeting people who have a higher risk of stroke. The strength of this study is that this was the first study that identifies the association between HKLS (Hypertension Knowledge Level Scale) and the awareness of stroke. Other studies did not use HKLS to explore the knowledge level of hypertension; they rather explore more about the knowledge level of stroke warning signs/symptoms or stroke risk factors. In addition, we also found that low HKLS (low knowledge of hypertension) contributed to low awareness of stroke. To our knowledge, this was also the first to report.

Nevertheless, this study has some limitations. First, we used one-item self-reported questionnaire to measure the awareness of being at risk of stroke. This may not be enough to validate the level of awareness. Therefore, future studies should elaborate the questionnaire with other more valid parameters to measure the level of stroke awareness. Second, the investigation related to their knowledge of stroke warning signs/symptoms was not further explored. Third, the participants in this study were recruited only from PHCs in Banyumas Regency in Indonesia. Therefore, the findings need careful interpretation for generalization. Finally, we only used a cross-sectional design. Thus, we could not provide the causal relationship between the awareness of stroke and the determinant factors although the direction of the association was consistent with prior studies.

## 5. Conclusions

Majority of hypertensive patients were unaware of being at risk of stroke. Knowledge of hypertension, income, and history of previous stroke are factors that may influence the awareness of being at risk of stroke among them. This emphasizes the need to provide better campaigns and education programs to raise the awareness of stroke in a community setting, particularly for the population at risk.

## Figures and Tables

**Table 1:** Characteristics of participants and its association with the awareness of stroke.

Characteristic	The awareness of stroke			*p*	OR	95% CI
	Low (*n*, %)	High (*n*, %)	Total (*n*, %)			
Sex						
Male	66 (14.5)	22 (4.8)	88 (19.3)	0.539		
Female	288 (63)	81 (17.7)	369 (80.7)		0.844	0.491-1.451
Age (years)						
<55	84 (18.4)	27 (5.9)	111 (24.3)	0.605		
≥55	270 (59.1)	76 (16.6)	346 (75.7)		0.876	0.530-1.448
Education level						
Low	283 (61.9)	81 (17.7)	364 (79.6)	0.773		
High	71 (15.5)	22 (4.8)	93 (20.3)		1.083	0.632-1.854
HKLS						
Low	211 (46.2)	45 (9.8)	256 (56)	0.004^∗^		
High	143 (31.3)	58 (12.7)	201 (44)		1.902	1.221-2.963
Married status						
Married	258 (56.5)	70 (15.3)	328 (71.8)	0.329		
Widowed	96 (21)	33 (7.2)	129 (28.2)		1.267	0.787-2.039
Living with						
Family	185 (40.5)	48 (10.5)	232 (51)	0.312		
Alone/couple	169 (37)	55 (12)	224 (49)		1.254	0.808-1.974
Income						
<MRP	252 (55.1)	55 (12)	307 (67.1)	0.001^∗^		
≥MRP	102 (22.3)	48 (10.5)	150 (32.8)		2.156	1.374-3.383

OR: odds ratio; CI: confidence interval; HKLS: Hypertension Knowledge Level Scale; MRP: minimal regional payment. ∗*p* < 0.05.

**Table 2:** Association between stroke risk factors with the awareness of stroke.

Stroke risk factors	The awareness of stroke			*p*	OR	95% CI
	Low (*n*, %)	High (*n*, %)	Total (*n*, %)			
Medication adherence						
No	209 (45.7)	61 (13.3)	270 (59)	0.973	0.992	0.635-1.551
Yes	145 (31.7)	42 (9.2)	187 (40.9)			
History of hypertension						
Controlled	45 (9.8)	22 (4.8)	67 (14.6)	0.029∗	0.536	
Uncontrolled	309 (67.6)	81 (17.7)	390 (85.3)			0.305-0.944
DM						
No	214 (46.8)	45 (9.8)	259 (56.6)	0.003∗	1.970	1.264-3.071
Yes	140 (30.6)	58 (12.7)	198 (43.4)			
Hyperlipidemia						
No	274 (60)	81 (17.7)	355 (77.7)	0.790	0.930	0.546-1.585
Yes	80 (17.5)	22 (4.8)	102 (22.3)			
History of prior stroke						
No	343 (75.1)	90 (19.7)	433 (94.8)	<0.001^∗^	4.504	
Yes	11 (2.4)	13 (2.8)	23 (5.2)			1.953-10.390
Atrial fibrillation						
No	314 (68.7)	91 (19.9)	405 (88.6)			
Yes	40 (8.8)	12 (2.6)	52 (11.4)	0.921	1.035	0.521-2.056
Heart failure						
No	336 (73.5)	94 (20.6)	430 (94.1)	0.166	1.787	0.778-4.108
Yes	18 (3.9)	9 (2)	27 (5.9)			
Sleep apnea						
No Yes	346 (75.7) 8 (1.8)	101 (22.1) 2 (0.4)	447 (97.8) 10 (2.2)	0.846	0.856	0.179-4.097

OR: odds ratio; CI: confidence interval; DM: diabetes mellitus. ^∗^*p* < 0.05; ^∗∗^*p* < 0.001.

**Table 3:** Multiple logistic regression of factors associated with low-level of stroke risk awareness after adjustment with covariates.

Variable	OR	95% CI	*p*
HK-LS (low)	1.942	1.195-3.158	0.007^∗^
Income (low)	2.321	1.326-4.064	0.003^∗^
History of stroke (no)	6.033	2.450-14.858	<0.001^∗∗^

OR: odds ratio; CI: confidence interval; HK-LS: Hypertension Knowledge Level Scale; ^∗^*p* < 0.05; ^∗∗^*p* < 0.001.

## Data Availability

The authors confirm that the data supporting the findings of this study are available within the article.
